# Assessment of Health Literacy and Self-reported Readiness for Transition to Adult Care Among Adolescents and Young Adults With Spina Bifida

**DOI:** 10.1001/jamanetworkopen.2021.27034

**Published:** 2021-09-28

**Authors:** James T. Rague, Soojin Kim, Josephine A. Hirsch, Theresa Meyer, Ilina Rosoklija, Jill E. Larson, Vineeta T. Swaroop, Robin M. Bowman, Diana K. Bowen, Earl Y. Cheng, Elisa J. Gordon, Daniel I. Chu, Tamara Isakova, Elizabeth B. Yerkes, David I. Chu

**Affiliations:** 1 Division of Urology, Ann & Robert H. Lurie Children’s Hospital of Chicago, Chicago, Illinois; 2 Department of Urologic Sciences, Faculty of Medicine, University of British Columbia, Vancouver, British Columbia, Canada; 3 Division of Orthopedic Surgery, Ann & Robert H. Lurie Children’s Hospital of Chicago, Chicago, Illinois; 4 Division of Neurosurgery, Ann & Robert H. Lurie Children’s Hospital of Chicago, Chicago, Illinois; 5 Center for Health Services and Outcomes Research, Institute for Public Health and Medicine, Northwestern University Feinberg School of Medicine, Chicago, Illinois; 6 Division of Transplantation, Northwestern University Feinberg School of Medicine, Chicago, Illinois; 7 Division of Gastrointestinal Surgery, University of Alabama at Birmingham, Birmingham; 8 Center for Translational Metabolism and Health, Institute for Public Health and Medicine, Northwestern University Feinberg School of Medicine, Chicago, Illinois; 9 Division of Nephrology and Hypertension, Northwestern University Feinberg School of Medicine, Chicago, Illinois

## Abstract

**Importance:**

Health literacy has been shown to play an important role in transitions of care in adult populations, with low health literacy associated with adverse health outcomes. The role of health literacy in the transition from pediatric to adult care has been less well studied. Among adolescents and young adults with spina bifida, high rates of unsuccessful transition have been shown, but how patient health literacy affects transition readiness remains unknown.

**Objective:**

To determine whether health literacy is associated with transition readiness in adolescents and young adults with spina bifida.

**Design, Setting, and Participants:**

This cross-sectional study involved collection of patient-reported questionnaires between June 2019 and March 2020 at a multidisciplinary spina bifida center at a single, free-standing children’s hospital. Patient demographic and clinical characteristics were obtained from medical record review. Patients were aged 12 years or older with a diagnosis of spina bifida (myelomeningocele and nonmyelomeningocele) whose primary language was English or Spanish. Data analysis was performed from October 2020 to March 2021.

**Exposures:**

Health literacy as assessed by the Brief Health Literacy Screening Tool.

**Main Outcomes and Measures:**

The primary outcome was total Transition Readiness Assessment Questionnaire (TRAQ) score, normalized into units of SD. Nested, multivariable linear regression models assessed the association between health literacy and TRAQ scores.

**Results:**

The TRAQ and Brief Health Literacy Screening Tool were completed by 200 individuals (median [range] age, 17.0 [12.0-31.0] years; 104 female participants [52.0%]). Most of the patients were younger than 18 years (110 participants [55.0%]) and White (136 participants [68.0%]) and had myelomeningocele (125 participants [62.5%]). The mean (SD) TRAQ score was 3.3 (1.1). Sixty-six participants (33.0%) reported inadequate health literacy, 60 participants (30.0%) reported marginal health literacy, and 74 participants (37.0%) reported adequate health literacy. In univariable analysis, health literacy, age, type of spina bifida, level of education, self-administration vs completion of the questionnaires with assistance, ambulatory status, and urinary incontinence were associated with total TRAQ score. In all nested, sequentially adjusted, multivariable models, higher health literacy remained a significant, stepwise, independent variable associated with higher TRAQ score. In the fully adjusted model, having adequate compared with inadequate health literacy was associated with an increase in normalized TRAQ score of 0.49 SD (95% CI, 0.19-0.79).

**Conclusions and Relevance:**

Patient-reported transition readiness is associated with health literacy, even after adjustment for education level and other demographic and clinical factors. Developing and implementing health literacy–sensitive care programs during the transition process may improve patient transition readiness.

## Key Points

**Question:**

Is health literacy associated with patient-reported care transition readiness among adolescents and young adults with spina bifida?

**Findings:**

In this cross-sectional, single-center study, 200 individuals completed patient-reported questionnaires between June 2019 and March 2020 during visits to a multidisciplinary pediatric spina bifida center. Health literacy was associated with care transition readiness even after adjusting for patient demographic and clinical characteristics.

**Meaning:**

These findings suggest that efforts should be directed to providing relevant health education to adolescents and young adults with spina bifida.

## Teaser

This cross-sectional study examines whether health literacy is associated with readiness to transition to adult care among adolescents and young adults with spina bifida.

## Introduction

Spina bifida (SB) encompasses various congenital disorders of the spinal cord and affects an estimated 1600 new births per year in the US (30 cases per 100 000 live births).^1^ The survival of infants born with SB has improved over time, with an estimated 75% to 90% now surviving into adulthood.^2^ Given the complexity of the disease, lifelong management of SB is associated with high rates of costly and possibly preventable health care utilization.^3,4,5^ To improve health and comprehensive care into adulthood, the transition from pediatric to adult SB care has been the subject of much discussion.^6,7,8,9,10,11^

Transition is defined as “the purposeful, planned movement of adolescents and young adults with chronic physical and medical conditions from child-centered to adult-oriented health-care systems.”^12^ Although the timing of transition has often centered on age, studies^13,14,15^ suggest that timing should instead center on readiness to navigate the adult health care system. Successful transition requires patient acquisition of skills surrounding health-related decision-making, self-care, and self-advocacy, which may not be age dependent.^16^ Prior research using the Transition Readiness Assessment Questionnaire (TRAQ)^17,18^ in patients aged 18 years and older with SB showed that age and female sex were associated with patient-reported transition readiness.^19,20^ Less is known about younger patients’ transition readiness or other potentially modifiable factors associated with transition readiness. In other chronic conditions, such as chronic kidney disease, rheumatological disease, and inflammatory bowel disease, health literacy (HL) has been associated with transition readiness.^21,22,23^ Similarly, HL has been shown to be an important factor in successful transitions of care in adult populations.^24^

HL is defined as “the degree to which individuals can obtain, process, and understand the basic health information and services they need to make appropriate health decisions.”^25^ Overall worse health outcomes in adult patients have been associated with poor HL.^26,27,28^ In the SB population, cognitive, neurobehavioral, and executive functional impairments, including aspects of literacy, are well described.^29,30,31,32^ Adolescent patients with SB and their caregivers have reported decreased HL in prior studies.^33,34^ However, to our knowledge, no research has examined whether HL is directly associated with transition readiness. This study assesses whether patient-reported HL in adolescents and young adults with SB is associated with transition readiness. We hypothesize that having adequate HL is associated with greater transition readiness.

## Methods

### Study Design and Patient Population

The Ann & Robert H. Lurie Children’s Hospital institutional review board granted approval for retrospective review of patient health records in this cross-sectional study. The study was exempt from the requirement of individual patient consent because the institutional review board determined that retrospectively collected patient data were deidentified, and the questionnaires were collected as part of standard clinical practice. This study followed the Strengthening the Reporting of Observational Studies in Epidemiology (STROBE) reporting guideline.^35^

Between June 2019 and March 2020, all patients aged 12 years or older with any form or degree of SB who were seen in our multidisciplinary SB center, which is part of a free-standing children’s hospital, were approached at the time of their clinic visits by a clinical research coordinator to complete various patient-reported questionnaires as part of the standard of care pathway. Questionnaire administration occurred at their regular visits with data incorporated into the medical record for clinical care and decision-making. Questionnaires were either self-administered, completed with assistance, or completed by a proxy (eg, parents, caregivers) if patients had insufficient cognitive capacity for self-administration. The care team familiar with eligible patients, with agreement from proxy (if present), determined capacity for self-administration. Those who acted as a proxy were directed to answer questions from the perspective of the patient. English and Spanish language questionnaires were available on the basis of the patient’s primary language. Patients younger than 12 years or those whose primary language was not English or Spanish were excluded. For this study, patients were further excluded if they did not complete the TRAQ and Brief Health Literacy Screening Tool (BRIEF)^36^ measures in full.

### Measures

The primary outcome was transition readiness measured by total TRAQ score. TRAQ is a non–disease-specific, 20-item validated questionnaire designed to assess patient perceived transition readiness with questions addressing the following domains: Managing Medications, Appointment Keeping, Tracking Health Issues, Talking With Providers, and Managing Daily Activities.^17,18^ Each question is scored on a 5-point Likert scale. A score of 1 represents not ready to transition at all, and a score of 5 represents fully ready to transition. Total TRAQ score is calculated as the mean of each domain score (score range, 1-5). Six patients left their clinic visit before completion of the full TRAQ questionnaire, and the questionnaire was completed at a subsequent visit within 6 months. In lieu of a predefined and validated clinically meaningful change in TRAQ score, we used 1 SD from the mean population TRAQ score as a surrogate for a minimal clinically important difference for the outcome measure.

The primary exposure was HL measured by the BRIEF score.^36^ BRIEF is a validated, 4-item screening tool to assess HL and is scored on a 5-point Likert scale per question with total summed scores ranging from 4 to 20. Total summed scores are categorized as inadequate HL (score, 4-12), marginal HL (score, 13-16), and adequate HL (score, 17-20).

### Covariates

Demographic data and clinical characteristics for each patient were extracted from the medical record. If all data points were not accessible on the basis of the clinic visit associated with the day of questionnaire completion, visits within 6 months of that date were assessed. Demographic and clinical variables were determined a priori according to existing data and factors thought to be associated with transition readiness. Demographic variables included age (<18 or ≥18 years), race (Asian, Black or African American, multiracial, other [specified as a category in the medical record with no free-text option], or White), ethnicity (Hispanic or Latino vs not Hispanic or Latino), type of insurance (private, public, or military), sex, and highest level of education achieved. Race and ethnicity were assessed because of their known associations with health literacy. For those individuals currently in primary or secondary school, the use of an individualized education plan (IEP) was determined according to documentation in the medical record. Clinical characteristics included type of spina bifida (eg, myelomeningocele [MM], lipomyelomeningocele, or fatty filum), presence of a ventricular shunt, ambulatory status based on the Hoffer classification^37^ (community, household, therapeutic, or nonambulator), lower extremity functional level of lesion measured by manual muscle test, primary bladder management strategy (eg, volitional voiding or clean intermittent catheterization [CIC]), and presence of any bladder or bowel incontinence (self-reported, at least 1 episode per month vs never). For patients who managed their bladder with CIC, we assessed whether CIC was performed independently or by a caregiver and whether a catheterizable channel was used, surmising that greater independence with bladder management would be associated with higher readiness to transition.

### Statistical Analysis

Descriptive statistics with proportions were calculated. Demographic and clinical variables were stratified by HL category, and χ^2^ tests were performed to assess variation across HL categories. Total TRAQ score was calculated as a continuous variable (histogram shown in eFigure in the Supplement). BRIEF scores were categorized as inadequate HL, marginal HL, or adequate HL. Several categorical variables were dichotomized for data reduction, including race (White vs all others), insurance status (private vs public or military), type of spina bifida (MM vs non-MM), level of education (primary or secondary school vs any level beyond secondary), and ambulatory status (ambulatory vs nonambulatory). Parametric statistics (*t* test and analysis of variance tests) were used in univariable analyses for the outcome of total TRAQ score.

Multivariable nested linear regression was performed to fit associations between BRIEF score and TRAQ score. The raw TRAQ score was transformed to a normalized score, such that a β coefficient of 1 represented a change in TRAQ score by 1 SD of the mean TRAQ score. A sequential approach to building the regression models was taken with the addition of demographic characteristics to BRIEF, then adding mobility and functional factors to the prior model, then education factors, and finally bowel and bladder factors. Regression coefficients for HL were determined with 95% CIs. *R*^2^ values from each model were obtained to assess how much of the variability of the TRAQ score was explained by the model.

A secondary analysis was performed to assess for effect modification of patient age category on the association between HL and TRAQ score. An interaction term between age and HL variables was included in the fully adjusted model. Significance of the addition of the interaction term was confirmed by a likelihood ratio test.

Two subgroup analyses were performed for individuals who managed their bladder with CIC and for individuals who were currently enrolled in school. The additional variables relating to those categories, as described already, were added to the linear regression model with all other covariates present. No interaction terms were included.

All results were considered statistically significant at a 2-tailed *P* < .05. Statistical analysis was performed using Stata statistical software version 16.1 (StataCorp). Data analysis was performed from October 2020 to March 2021.

## Results

The TRAQ and BRIEF were completed by 200 patients (median [range] age, 17.0 [12.0-31.0] years; 104 female participants [52.0%]). Most of the patients were younger than 18 years (110 participants [55.0%]) and White (136 participants [68.0%]) and had MM (125 participants [62.5%]). A total of 241 eligible patients presented to clinic during the study period, of whom 232 (96.3%) completed questionnaires and 200 of 232 (86.2%) completed both the TRAQ and BRIEF questionnaires in their entirety and were included in this analysis. Complete demographic and clinical characteristics of the study cohort, stratified by HL category, are presented in Table 1. On the basis of BRIEF scores, 66 participants (33.0%) reported inadequate HL, 60 participants (30.0%) reported marginal HL, and 74 participants (37.0%) reported adequate HL. Comparison of the included cohort against those who did not complete the questionnaires showed no significant differences in baseline characteristics, except for the latter group having more female participants, fewer participants in school, fewer participants who self-administered the questionnaires, more participants with higher lesion levels, and fewer participants who volitionally void for bladder management.

**Table 1.** Demographic and Clinical Characteristics of Individuals With Spina Bifida Who Completed Transition Readiness Assessment Questionnaire and Brief Health Literacy Screening Tool Stratified by Health Literacy Category

Characteristics	Participants, No. (%) (N = 200)				*P* value
	Total population	Health literacy category			
		Inadequate	Marginal	Adequate	
Age, y					
<18	110 (55.0)	43 (36.1)	32 (29.1)	35 (31.8)	.10
≥18	90 (45.0)	23 (25.6)	28 (31.1)	39 (43.3)	
Race					
Asian	12 (6.0)	1 (8.3)	5 (41.7)	6 (50.0)	.29
Black	23 (11.5)	10 (43.5)	9 (39.1)	4 (17.4)	
Multiracial	4 (2.0)	2 (50.0)	1 (25.0)	1 (25.0)	
Other^a^	25 (12.5)	10 (40.0)	8 (32.0)	7 (28.0)	
White^b^	136 (68.0)	43 (31.6)	37 (27.1)	56 (41.2)	
Ethnicity					
Hispanic or Latino	68 (34.0)	25 (36.8)	24 (35.3)	19 (27.9)	.16
Non-Hispanic or non-Latino	132 (66.0)	41 (31.1)	36 (27.3)	55 (41.7)	
Insurance					
Private	112 (56.0)	31 (27.7)	28 (25.0)	53 (47.3)	.003
Public	84 (42.0)	35 (41.7)	29 (34.5)	20 (23.8)	
Military	4 (2.0)	0	3 (75.0)	1 (25.0)	
Sex					
Male	96 (48.0)	26 (27.1)	30 (31.3)	40 (41.7)	.21
Female	104 (52.0)	40 (38.5)	30 (28.9)	34 (32.7)	
Type of spina bifida					
Myelomeningocele	125 (62.5)	49 (39.2)	37 (29.6)	39 (31.2)	.001
Fatty or thickened filum or low-lying cord	34 (17.0)	14 (41.2)	7 (20.6)	13 (38.2)	
Lipomyelomeningocele	34 (17.0)	0	14 (41.2)	20 (58.8)	
Terminal myelocystocele	4 (2.0)	2 (50.0)	2 (50.0)	0	
Split cord malformation	3 (1.5)	1 (33.3)	0	2 (66.7)	
Highest level of education					
Primary or secondary school	143 (71.5)	59 (41.2)	44 (30.8)	40 (28.0)	<.001
Some college or technical school	43 (21.5)	0	13 (30.2)	24 (55.8)	
College degree	11 (5.5)	0	2 (18.2)	9 (81.8)	
Technical school graduate	2 (1.0)	0	1 (50.0)	1 (50.0)	
Other	1 (0.5)	1 (100.0)	0	0	
Currently in school?^c^					
Yes	149 (74.5)	53 (35.6)	44 (29.5)	52 (34.9)	.39
No	51 25.5)	13 (25.5)	16 (31.4)	22 (43.1)	
Does the student have an individualized education plan? (n = 126)^d^					
Yes	60 (47.6)	33 (55.0)	16 (26.7)	11 (18.3)	<.001
No	66 (52.4)	12 (18.2)	21 (31.8)	33 (50.0)	
Who completed the questionnaires?					
Self-administered	127 (63.5)	20 (15.8)	46 (36.2)	61 (48.0)	<.001
With assistance^e^	73 (36.5)	46 (63.0)	14 (19.2)	13 (17.8)	
Does the patient have a ventricular shunt?^f^					
Yes	100 (50.0)	44 (44.0)	25 (25.0)	31 (31.0)	.004
No	100 (50.0)	22 (22.0)	35 (35.0)	43 (43.0)	
Ambulatory status (Hoffer-classification)					
Community ambulator	152 (76.0)	40 (26.3)	49 (32.2)	63 (41.5)	.01
Household ambulator	10 (5.0)	5 (50.0)	2 (20.0)	3 (30.0)	
Therapeutic ambulator	3 (1.5)	3 (100.0)	0	0	
Nonambulator	35 (17.5)	18 (51.4)	9 (25.7)	8 (22.9)	
Lower extremity functional level					
Thoracic	35 (17.5)	17 (48.6)	10 (28.6)	8 (22.9)	.13
Lumbar	79 (39.5)	27 (34.2)	24 (30.4)	28 (35.4)	
Sacral	86 (43.0)	22 (25.6)	26 (30.2)	38 (44.2)	
Primary bladder management strategy					
Volitional void	54 (27.0)	14 (25.9)	14 (25.9)	26 (48.2)	.39
Clean intermittent catheterization	141 (70.5)	49 (34.8)	45 (31.9)	47 (33.3)	
Vesicostomy	1 (0.5)	1 (0.5)	0	0	
No management or elective incontinence	4 (2.0)	2 (50.0)	1 (25.0)	1 (25.0)	
Who performs clean intermittent catheterization? (n = 141)					
Patient	127 (90.1)	39 (30.7)	44 (34.7)	44 (34.7)	.008
Parent or caregiver	14 (9.9)	10 (71.4)	1 (7.1)	3 (21.4)	
Does the patient have a catheterizable channel? (n = 141)					
Yes	37 (26.2)	9 (24.3)	15 (40.5)	13 (35.1)	.25
No	104 (73.7)	40 (38.5)	30 (28.9)	34 (32.7)	
Any degree of bladder incontinence reported?					
Yes	93 (46.5)	38 (40.9)	28 (30.1)	27 (29.0)	.04
No	107 (53.5)	28 (26.2)	32 (29.9)	47 (43.9)	
Any degree of bowel incontinence reported?					
Yes	57 (28.5)	24 (42.1)	16 (28.1)	17 (29.8)	.20
No	143 (71.5)	42 (29.4)	44 (30.8)	57 (39.9)	

^a^ All 25 participants identified as Hispanic or Latino ethnicity. Other was specified as a category with no free-text option.

^b^ Forty-three participants identified as Hispanic or Latino ethnicity.

^c^ Includes all levels of education.

^d^ Applies only to primary or secondary school, and data were not available for all patients currently in school (n = 126).

^e^ Includes patient participating with assistance and completion by a patient proxy.

^f^ Includes ventriculoperitoneal, ventriculoatrial, and ventriculopleural shunts.

Distributions of TRAQ scores were calculated for each individual characteristic and are presented in Table 2. The overall mean (SD) TRAQ score was 3.3 (1.1), suggesting that patients were learning the skills necessary to transition.^18^ In univariable analysis, total TRAQ score was associated with age, type of SB, level of education, schooling status, presence of an IEP, self-administration vs completion of the questionnaires with assistance, ambulatory status, lower extremity functional level, who performs CIC, and urinary continence status. Race, ethnicity, insurance status, sex, and ventricular shunt status were not statistically significantly associated with total TRAQ score. Higher TRAQ scores were associated with higher HL (1-way analysis of variance, *F*_2,197_ = 23.81; *P* < .001) (Figure).

**Table 2.** Distribution of TRAQ Scores Based on Individual’s Characteristics

Characteristics	Total TRAQ score, mean (SD)	*P* value^a^
Total population (N = 200)	3.2 (1.1)	Not applicable
Age group, y		
<18	2.8 (0.9)	<.001
≥18	3.8 (1.0)	
Race		
White	3.2 (1.2)	.10
All others	3.5 (0.9)	
Ethnicity		
Hispanic or Latino	3.4 (1.1)	.16
Non-Hispanic or non-Latino	3.2 (1.1)	
Insurance		
Private	3.3 (1.1)	.81
Other	3.3 (1.1)	
Sex		
Male	3.3 (1.1)	.87
Female	3.3 (1.1)	
Type of spina bifida		
Myelomeningocele	3.2 (1.1)	.05
Nonmyelomeningocele	3.5 (1.0)	
Level of education		
Primary or secondary school	2.9 (1.0)	<.001
Any level of education beyond secondary	4.1 (0.8)	
Currently in school?		
Yes	3.1 (1.0)	<.001
No	3.8 (1.1)	
Does individual have an individualized education plan?		
Yes	2.6 (1.0)	<.001
No	3.5 (1.0)	
Who completed the questionnaires?		
Self-administered	3.6 (0.9)	<.001
With assistance	2.6 (1.1)	
Does the patient have a ventricular shunt?		
Yes	3.2 (1.1)	.17
No	3.4 (1.1)	
Ambulatory status		
Nonambulatory	2.9 (1.2)	.01
Ambulatory	3.4 (1.1)	
Lower extremity functional level		
Thoracic	3.0 (1.2)	.02
Lumbar	3.2 (1.1)	
Sacral	3.5 (1.0)	
Primary bladder management strategy		
Volitional void	3.4 (1.0)	.77
Clean intermittent catheterization	3.2 (1.1)	
Vesicostomy (n = 1)	3.0	
No management or elective incontinence	2.9 (1.3)	
Who performs clean intermittent catheterization?		
Patient	3.4 (1.1)	<.001
Parent or caregiver	2.2 (1.2)	
Does the patient have a catheterizable channel?		
Yes	3.1 (1.2)	.44
No	3.3 (1.1)	
Any degree of bladder incontinence reported?		
Yes	3.1 (1.0)	.02
No	3.4 (1.1)	
Any degree of bowel incontinence reported?		
Yes	3.1 (1.2)	.25
No or unknown	3.3 (1.1)	

Abbreviation: TRAQ, Transition Readiness Assessment Questionnaire.

^a^ *P* values were calculated by use of the *t* test or analysis of variance.

**Figure.**
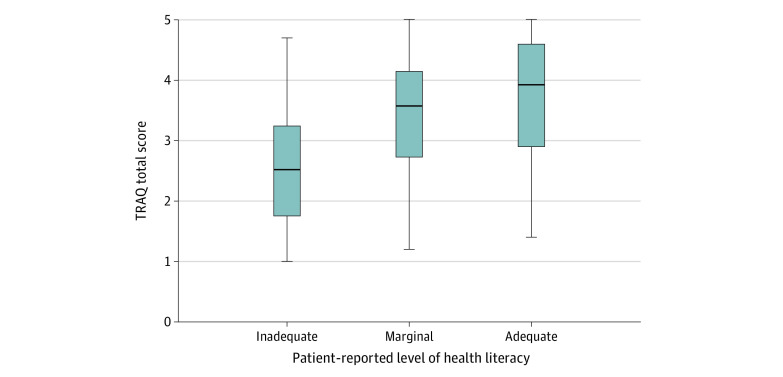
Variation in Total Transition Readiness Assessment Questionnaire (TRAQ) Score Based on Patient Reported Level of Health Literacy (Brief Health Literacy Screening Tool Score) Lines in center of boxes denote medians, tops and bottoms of boxes denote IQRs, and error bars denote upper and lower adjacent values.

In multivariable analyses, HL remained significantly associated in a stepwise manner with TRAQ score, even as covariates were added in sequential models (Table 3). However, the β coefficients in the final model failed to exceed the 1 SD value denoting a minimal clinically important difference. In model 1 with BRIEF alone, compared with inadequate HL, having adequate HL was associated with an estimated increase in normalized TRAQ score of 1.03 SD (95% CI, 0.73-1.33), and marginal HL was associated with an estimated increase of 0.76 SD (95% CI, 0.44-1.07). Nearly 20% of the variation in TRAQ score was explained by BRIEF alone. In model 5, the fully adjusted model, compared with those with inadequate HL, having adequate HL was associated with an estimated increase in normalized TRAQ score of 0.49 SD (95% CI, 0.19-0.79), and having marginal HL was associated with an estimated increase of 0.36 SD (95% CI, 0.06-0.65). The final adjusted model explained 50% of the variability in total TRAQ score. In the final model, older age, race other than White, self-administration vs completion of the questionnaires with assistance, and any education beyond secondary education were also significantly associated with a higher normalized TRAQ score.

**Table 3.** Multivariable, Nested Linear Regression Models of the Association Between HL and Normalized Transition Readiness Score Among 200 Participants

Linear regression model^a^	Inadequate HL, β (95% CI)^b^	Marginal HL, β (95% CI)^b^	*P* value	Adequate HL, β (95% CI)^b^	*P* value	Model *R*^2^ value
Model 1^c^	1 [Reference]	0.76 (0.44-1.07)	<.001	1.03 (0.73-1.33)	<.001	0.19
Model 2^d^	1 [Reference]	0.61 (0.33-0.84)	<.001	0.86 (0.50-1.05)	<.001	0.40
Model 3	1 [Reference]	0.55 (0.27-0.93)	<.001	0.78 (0.55-1.16)	<.001	0.44
Model 4	1 [Reference]	0.36 (0.07-0.65)	.02	0.49 (0.20-0.79)	.001	0.49
Model 5^e^	1 [Reference]	0.36 (0.06-0.65)	.02	0.49 (0.19-0.79)	.001	0.50

Abbreviation: HL, health literacy.

^a^ Each model iteration represents the addition of confounding variables, keeping all variables in previous model.

^b^ β coefficient of 1 represents a change in Transition Readiness Assessment Questionnaire score of 1 SD from the mean.

^c^ Model 1 includes Brief Health Literacy Screening Tool score only.

^d^ Model 2 includes the addition of individual demographic variables (eg, age, sex, race, and spina bifida type).

^e^ Model 5 includes the addition of bladder and bowel factors (eg, primary bladder management strategy, bladder incontinence, and bowel incontinence).

In our secondary analysis, the association between HL and TRAQ score did show effect modification by patient age category in the fully adjusted model (eTable in the Supplement). In patients aged 18 years or older, compared with inadequate HL, having adequate HL was associated with an estimated increase in normalized TRAQ score of 1.01 SD (95% CI, 0.55-1.46) and marginal HL was associated with an increase of 0.71 SD (95% CI, 0.26-1.16). In patients younger than 18 years, HL was no longer significantly associated with TRAQ score in the fully adjusted model. In 2 subgroup analyses for the 126 individuals who were enrolled in school at the time of questionnaire completion and the 141 participants who managed their bladder with CIC, HL remained significantly associated with TRAQ score, except in adolescents currently in school who had marginal HL compared with inadequate HL (Table 4).

**Table 4.** Subgroup Analyses Assessing Associations Between HL and Normalized Transition Readiness Score

Regression model	Inadequate HL, β (95% CI)^a^	Marginal HL, β (95% CI)^a^	*P* value	Adequate HL, β (95% CI)^a^	*P* value	Model *R*^2^ value
Individuals in school (n = 126)	1 [Reference]	0.34 (−0.05 to 0.73)	.09	0.42 (0.02 to 0.81)	.04	0.45
Individuals who use clean intermittent catheterization (n = 141)	1 [Reference]	0.37 (0.04 to 0.72)	.03	0.65 (0.31 to 1.00)	<.001	0.57

Abbreviation: HL, health literacy.

^a^ Coefficients are from the final adjusted multivariable model (variables present in model 5 in Table 3) with the inclusion of individualized education plan status for the individuals in school model and the addition of catheterizable channel status and who cleans intermittent catheterization (patient alone vs with assistance) for the individuals who clean intermittent catheterization model. A β coefficient of 1 represents a change in Transition Readiness Assessment Questionnaire score of 1 SD from the mean score.

## Discussion

In this cross-sectional study of adolescents and young adults with SB, we found that higher patient-reported HL was independently and persistently associated in a dose-dependent manner with statistically significant increases in patient-reported transition readiness, even after adjusting for numerous demographic and clinical characteristics. However, these increases in transition readiness failed to meet our 1 SD definition of a minimal clinically important difference. By itself, patient-reported HL measured by BRIEF accounted for 20% of the variability in TRAQ scores. Our findings suggest that evaluating HL may be a valuable component of the transition readiness assessment. Improving an individual’s transition readiness and ultimately improving transition success may be possible through implementation of more HL-sensitive care programs.

The ideal transition readiness assessment for patients with SB is not well defined, but a variety of measurement tools exist, such as TRAQ. However, TRAQ does not directly identify modifiable patient, social, or system factors for practitioners to use to improve patients’ transition readiness. Thus, understanding the patient factors associated with TRAQ scores in patients with SB is important. Other studies have attempted to assess this, but they excluded a measure of the ability to navigate the health care system based on HL.^19^ In our analyses, we identified that patient-reported HL remained significantly associated with TRAQ score even after adjustment for all covariates, including education factors. Although the magnitude of the association was attenuated, HL was still independently associated with TRAQ score in a stepwise manner. Furthermore, subgroup analyses in those patients who performed CIC and those who were in school also showed that HL was significantly associated with TRAQ score after additional adjustment for measures of patient independence and intellectual ability (ie, IEP status), respectively.

The association between patient-reported HL and transition readiness has not been previously well studied in patients with SB. Lightfoot et al^34^ showed that HL was lower among adolescents with SB compared with individuals without SB, but they did not test associations with transition readiness. Others have assessed the association between HL and transition readiness in non-SB chronic conditions, including childhood rheumatic disease, chronic kidney disease or hypertension, and inflammatory bowel disease, and reported findings similar to those in the present study.^21,22,23^ Zhong et al^21^ demonstrated a positive association between HL and self-management skills, communication with practitioners, and overall transition readiness in patients with chronic kidney disease and hypertension.

More broadly, patient-reported HL has been consistently shown to be significantly associated with other various health outcomes. Low HL in the general population is associated with poor self-efficacy, exacerbation of health problems, and increased health care expenditures.^38^ There is increasing recognition that HL plays a role in surgical outcomes,^39^ which has important implications in patients with SB who require frequent operations. Independently navigating the health care system and participating in health care decision-making requires a threshold level of knowledge and intellectual ability. The 2003 National Assessment of Adult Literacy demonstrated that only 12% of adults surveyed had proficient, task-based HL.^40^ Higher HL scores were associated with higher self-reported overall health. Although it is not a solution, recognition of low HL may be a pivotal step in improving long-term health outcomes and health care cost in patients with SB.

Various strategies to intervene for patients with low HL have been studied. Promoting HL in youth has been proposed, including institution of HL curricula in public schools and school nurse–guided fostering of HL in school-aged children and their parents.^41,42^ Another strategy entails changing health care systems to accommodate patients of varied HL levels through patient-centered communication, appropriate health education materials, and tailored self-management support systems.^43^ Low concordance between physician-perceived and patient-reported HL have been shown in adult populations.^44^ Inadequate or lack of assessment of HL in adolescents may lead to physician perception that patients have a better understanding than they do. This assumption may lead unprepared patients into the adult health care system where less support is available. When low HL is identified early, a greater focus on parental and family needs can drive transition planning.

One common clinical question is when to introduce the concept of transitioning care to pediatric patients. Biological age alone is neither a suitable nor generalizable cutoff to transfer patients to adult care settings. A study using group-based trajectory modeling of medical self-responsibility among 140 youth with SB found that one-third of the cohort never reached shared self-responsibility with caregivers even by ages 16 to 17 years old, still requiring mostly caregiver responsibility.^45^ In 2018, an updated multicollaborative clinical report discussed 6 core elements necessary for successful health care transition.^46^ Although it was not specific to patients with SB, the report highlighted the importance of formal readiness assessment between ages 14 and 18 years old. The 2018 Guidelines for the Care of People with Spina Bifida recommend beginning this process earlier (age 12 years) because of the potential for increased difficulty with self-management and time needed for appropriate planning and skill acquisition.^47^ In our SB center, we start embedding measures, including TRAQ, for all patients aged 12 years or older, as part of our clinical standard of care.

Understanding that greater HL is associated with greater transition readiness provides us with an opportunity to improve care within our SB center. Quality improvement projects aimed at addressing HL-sensitive education materials, individualized transition plans accommodating a patient’s HL-specific needs adjusting for cognitive limitations, repeat measures over time, and further research into the association of HL, transition readiness, and successful completion of transition to our adult clinic are required.

### Strengths and Limitations

Our study has strengths in that it is one of the first studies to assess the association between HL and transition readiness in patients with SB. We obtained a diverse patient sample with demographic and clinical characteristics similar to those of the larger population living with SB as reported by the National Spina Bifida Patient Registry.^48,49^

Our study also has limitations. This is a single-center study with limited generalizability and possible selection bias. Unmeasured confounders may exist, such as physician-, family-, and system-level factors known to be important barriers to successful transition^9,45^ or the length of time each patient had been in our care before completion of questionnaires. However, we attempted to control for multiple known factors, including some specific potential factors not previously studied, such as IEP use (as an imperfect surrogate for intellectual ability) or need for assistance in completing the questionnaires. The administration of TRAQ and BRIEF was modified by allowing parental or caregiver assistance with completion. Although TRAQ was designed to assess transition readiness in youth with chronic conditions, it is not validated in the SB population specifically, is self-reported, leaving room for bias, and lacks evidence of association with better health outcomes or quality of life. However, TRAQ may identify specific skills that can be targeted. There is also no validation of what constitutes a clinically significant incremental TRAQ score change, thus prompting us to use 1 SD as a surrogate. Although there is a TRAQ-SB now, this was published after our study period started.^50^ Similarly, BRIEF was validated in an adult, non-SB population and was designed for self-administration. Therefore, the validity of HL findings in individuals younger than 18 years with SB may be limited. Furthermore, our study design was cross-sectional, meaning that the association between HL and transition readiness does not imply causality.

## Conclusions

This study found that HL is associated with patient-reported transition readiness when controlling for demographic and clinical factors in adolescents and young adults with SB. Those with low HL reported being less ready to transition, which highlights an opportunity to improve transition readiness. Understanding this association will allow for future patient-centered and HL-based interventions to improve the process of transition to adult care.

## Notes

Supplement. **eTable.** Fully Adjusted Multivariable Linear Regression Model of the Association Between Health Literacy and Standardized Transition Readiness Score With Effect Modification by Patient Age Category, N=200 **eFigure.** Histogram of Raw Total TRAQ Scores, N=200
