# Supplementary material for: Assessment of Health Literacy and Self-reported Readiness for Transition to Adult Care Among Adolescents and Young Adults With Spina Bifida
Source: JAMA Netw Open. 2021 Sep 28;4(9):e2127034. doi: 10.1001/jamanetworkopen.2021.27034 (PMC8479582; doi:10.1001/jamanetworkopen.2021.27034)
Supplement: Supplement. — eTable. Fully Adjusted Multivariable Linear Regression Model of the Association Between Health Literacy and Standardized Transition Readiness Score With Effect Modification by Patient Age Category, N=200 eFigure. Histogram of Raw Total TRAQ Scores, N=200 [file jamanetwopen-e2127034-s001.pdf]

Supplemental Online Content

Rague JT, Kim S, Hirsch JA, et al. Assessment of health literacy and self-reported readiness for transition to adult care among adolescents and young adults with spina bifida. *JAMA Netw Open*. 2021;4(9):e2127034. doi:10.1001/jamanetworkopen.2021.27034

eTable. Fully Adjusted Multivariable Linear Regression Model of the Association Between Health Literacy and Standardized Transition Readiness Score With Effect Modification by Patient Age Category, N=200

eFigure. Histogram of Raw Total TRAQ Scores, N=200

This supplemental material has been provided by the authors to give readers additional information about their work.

© 2021 Rague JT et al. *JAMA Network Open.*

eTable. Fully Adjusted Multivariable Linear Regression Model of the Association Between Health Literacy and Standardized Transition Readiness Score With Effect Modification by Patient Age Category, N=200

**Patient Age-Group**	**Regression Co-efficient (95% CI)[a], p-value**		
	**Inadequate HL**	**Marginal HL**	**Adequate HL**
≥18yo group	Reference	0.71 (0.26-1.16), 0.002	1.01 (0.55-1.46), <0.001
<18yo group	Reference	0.19 (-0.16-0.55), 0.30	0.20 (-0.16-0.56), 0.26

a. Estimates were obtained with the addition of an interaction term between age and health literacy to the model. A beta co-efficient of 1 represents a change in TRAQ score of 1 standard deviation from the mean TRAQ score.

© 2021 Rague JT et al. *JAMA Network Open.*

eFigure. Histogram of Raw Total TRAQ Scores, N=200

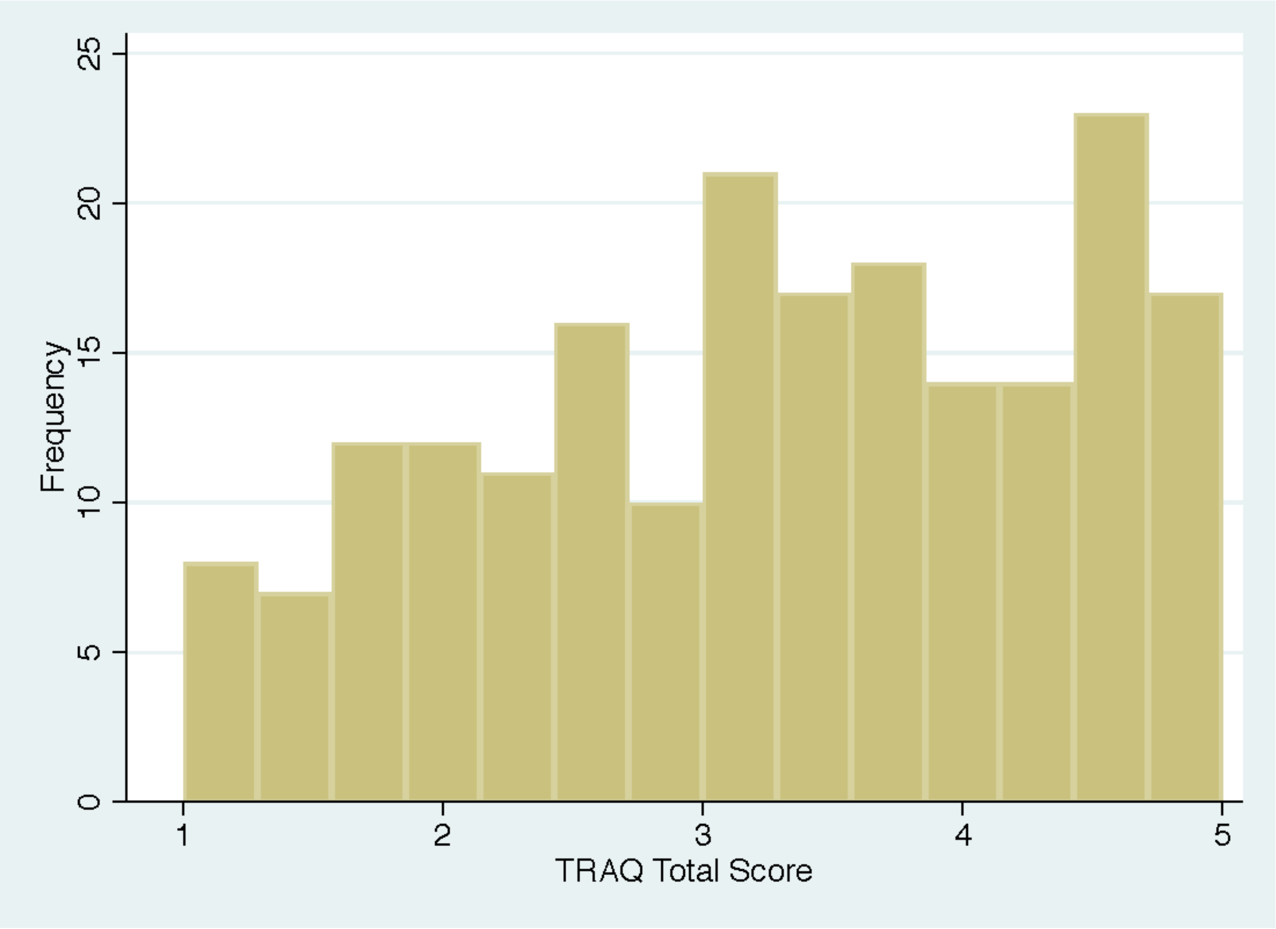

© 2021 Rague JT et al. *JAMA Network Open.*
